# Narrativity and enaction: the social nature of literary narrative understanding

**DOI:** 10.3389/fpsyg.2014.00895

**Published:** 2014-08-22

**Authors:** Yanna B. Popova

**Affiliations:** Department of Cognitive Science, Case Western Reserve UniversityCleveland, OH, USA

**Keywords:** narrative, narrative understanding, literature, participatory sense-making, social cognition

## Abstract

This paper proposes an understanding of literary narrative as a form of social cognition and situates the study of such narratives in relation to the new comprehensive approach to human cognition, enaction. The particular form of enactive cognition that narrative understanding is proposed to depend on is that of participatory sense-making, as developed in the work of Di Paolo and De Jaegher. Currently there is no consensus as to what makes a good literary narrative, how it is understood, and why it plays such an irreplaceable role in human experience. The proposal thus identifies a gap in the existing research on narrative by describing narrative as a form of intersubjective process of sense-making between two agents, a teller and a reader. It argues that making sense of narrative literature is an interactional process of co-constructing a story-world with a narrator. Such an understanding of narrative makes a decisive break with both text-centered approaches that have dominated both structuralist and early cognitivist study of narrative, as well as pragmatic communicative ones that view narrative as a form of linguistic implicature. The interactive experience that narrative affords and necessitates at the same time, I argue, serves to highlight the active yet cooperative and communal nature of human sociality, expressed in the many forms than human beings interact in, including literary ones.

## Setting the stage: how do narratives mean?

Stories are everywhere in human lives and storytelling is indeed part of all human cultures. We think in narrative, remember in narrative and interact in narrative. People tell stories in words, in pictures and in movement, in musical forms, and through increasingly diverse multimodal means. We learn through stories told in the news and in history books, we make decisions based on stories reported in criminal trials, we find it effortless to engage with the fictional stories revealed in our favorite novels and films. As the semiotician Barthes had noted, “narrative is international, transhistorical, transcultural: it is simply there like life itself” (Barthes, 1977, p. 79). The question remains, however: why and how are human experiences best organized by stories?

Stories have been studied for centuries from a variety of perspectives and with distinct questions in mind. Although a much scrutinized subject and the topic of many volumes, the field of narrative research is still an open one. That narratives play an irreplaceable role in human knowledge organization is undeniable, yet the reasons for that very fact remain elusive and ultimately dependent on the orientation of the research paradigm asking the questions. Most broadly, work on narrative can be divided between positivistic (scientific) and hermeneutic (humanistic) approaches, although that very division often cuts across individual disciplines and even theorists. Therefore, as I will argue in this article, narrative is best studied from the point of view of a new and emerging approach to the study of the mind as developed in the enactive paradigm. While cognitive science from its inception has aspired to represent the true marriage of humanistic and scientific ways of understanding, this merging of aims is only just beginning to be realized in what is termed “enactive cognitive science.” This article also attempts to frame some common research topics between the theoretical study of narrative, as undertaken historically, and current cognitive science. In a book length study (Popova, in press) I have developed a model of narrative understanding as a cognitive process reliant on perceptual causality, a phenomenon distinct from mere temporal succession, and experienced as inherently meaningful, thus linking it to the important work of Michotte and his intellectual descendants (Michotte, 1963). The experiential notion of perceptual causality is used to flesh out an understanding of narrative causality and our conception of action sequences in stories: their intentional nature and their telicity (the fact that they have beginnings and endings). This is in tune with a broadly phenomenological understanding of narrative as strongly implying a meaningful causal structuring, a teleological grasping of the events of a story in a particular way. This proposal goes toward explaining narrative's acknowledged ubiquity as a form of knowledge organization in a principally non-representationalist or functionalist way. Definitional in the enactive approach is that cognition bears a constitutive relation to its objects. In a similar vein, in my understanding story is defined further as a relational domain constituted or enacted in the very interaction between an autonomous agency responsible for the causal contingencies of the narrative and most commonly known as a narrator, and the reader. A recognition of the presence of such a narrating consciousness that relays the narrative events and thereby shapes them in the process of telling, and how the story develops in interaction with the reader, will be developed and explained through the notion of “participatory sense-making” as proposed and elaborated in the enactive view of human cognition (De Jaegher and Di Paolo, 2007).

## Intentionality in narrative understanding

Human lives are driven by living in a world where actions take both a practical and a theoretical priority. From the events of everyday life, to participation in cultural acts, to just being in the world, our primary way of interacting with a world is through practical action. Action is most commonly the result of coordinated movement but it is commonly accepted that not all movement constitutes an action. Most philosophers and others deliberating these problems would agree that it is human intention or purpose that transforms a movement into a deliberate action, the latter being understood as both the self-awareness of pursuing a specific goal, and the recognition by others that an agent's actions are also deliberate or goal-directed. As some phenomenologists have argued, the very experience of one's own intentionality is linked to the agent's own self-reflexive consciousness of agency: the awareness that I know that I can cause something to happen^1^. Such a phenomenology of agency that we possess and that we reciprocally understand others to possess has been plausibly linked to the evolutionary and cognitive advantages afforded to our ancestors by the ability to voluntarily control the body as a means of communicating meaning^2^. Using the body thus as an instrument or as a representational mechanism of sorts has been a means of providing our ancestors, but also any normally developing infant, with a bodily-based sense of agency. Accepting that human beings are regularly driven by intention and that intention is to some extent readable for the people that surround them and share their social and perceptual world leads also to another fundamental aspect of human consciousness. As understood in phenomenology, this view describes the understanding that all consciousness (all perceptions, imaginings, memories, etc) is intentional, it has directedness toward an object or person, it is “about or of something”^3^. Such an understanding immediately calls attention to an inevitable consequence of this, namely, that human thought is intrinsically tied to the world, be it in the form of physical objects or other living beings. This also means that human actions are always already understood by other human beings within a context of intention, motives and goals, and not as mere physical movements or random events. In the context of action, human movements are grasped together, holistically, as an action, or a series of actions. Our lived experience, as embodied creatures within a social world, is therefore intrinsically meaningful to ourselves and to others. Furthermore, a mere unreflective instinctive behavior is to be distinguished from true agency. Thus, my sitting on the computer with the intention to write an article is an action, but a bird's singing outside my window to attract a mate is better described as an instinctual response to a possible physiological need. The reason for this distinction is that my purpose to write an article may not be narrowed down to just one thing and thus may not be uniquely determined or understood by others and even by myself, covering instead a wide plethora of goals, motivations, and circumstances, all of which surpass by far an animal's more narrowly understood series of actions and their expected, because ultimately predetermined, outcome. Human agency thus covers many reasons for acting, which is precisely what cannot be said of non-human agents. What matters for human intentionality then, including how we understand it when applied to text interpretation, is that intention itself should not be understood as always uniquely determined or initially hidden and then discovered or discoverable, but as emerging from a process of interaction between agents.

The purpose of the above interlude has been to situate the discussion of narrative understanding that is to follow in the same context of agency, intentionality and dynamic interaction that have characterized more recent developments in the study of human action, perception and consciousness. In its initial description the enactive approach (Varela et al., 1991) emphasized the indelible link between cognitive processes and an organism's embedded activity. Sensorimotor enactivism, as subsequently developed in the work of Noë and colleagues (Noë, 2004, 2010; see also Hutto and Myin, 2013) explains the practical knowledge characteristic of perception, understood as a process of interaction between an organism and its environment. But social interactions, rather than sensorimotor ones, dominate certain human practices, specifically the production and reception of narratives. We act in the world in no small measure because we expect our actions and intentions to be understood as meaningful, to be made sense of, by other people. Human lives in all their inherent complexities take place in the open space of shared realities and shared meanings, not within individual isolated brains. More importantly still, while the agency of an individual is of great importance for sociality, it is acting for and through one another (interacting) that ultimately defines who we are. Our human world is a social world and it takes place in large measure outside of our brains, in the common shared activity that is life. If we take this view and apply it in a wider framework, as I will be doing currently, we can see the reading and understanding of books as essentially not that different from other forms of interaction within a social world: through a careful and deliberate process of intersubjective sense-making.

Existing characterizations of the reading process of fictional narratives foreground the nature of meaning in human communication in general, irrespective of disciplinary affiliation. How do narratives mean? How do readers make sense of written stories? How can this process be best described and explained? These are the questions guiding the research. There are many ways in which the reading of fiction has been theorized and studied mainly by literary scholars, but also by discourse specialists, psychologists and linguists. With some degree of simplification it can be stated that, despite their differences, the vast amount of existing approaches see narrative understanding as a process of communication in which the written text offers meaning and leads to interpretation through some degree of involvement on the part of the reader. These approaches can thus be classified as generally contributing to the explication of a process of “narrative transmission” between an addresser and an addressee in a given act of communication. From early literary theory (Jacobson, 1960), through speech-act theory (Searle, 1975) and relevance theory (Sperber and Wilson, 1995), to rhetoric (Booth, 1961), and studies of discourse (Graesser et al., 1994) literary communication has been assumed to take place between the multiple identities and functions of the person believed to be sending the message: “real author,” “implied author,” “narrator,” and the equally multiple assumed identities of the “addressee”: “real reader,” “implied reader,” “narratee.” Within this basic communicative set-up, many distinctions have been drawn with respect to the degree to which the process of narrative transmission is mainly text-centered or reception-centered, on the one hand, and who the main participants in the process itself are, on the other. I will deal with each of those distinctions briefly and under separate rubrics in the next few sections. My own hypothesis about narrative understanding as participatory sense making will be developed in Sections Narrative Enaction: Changing the Assumptions of Narrative Understanding, Narrative Enaction and Participatory Sense-Making, and Narrative Enaction: Current Empirical Data and Future Possibilities below.

## Narrative as inherent structure: text-centered approaches

In this group belong theories that seek to examine textual features, properties and characteristics of the narrative text itself as the most significant aspect of the meaning construal process. The definitional criteria of narrative proposed in formalist and structuralist theories have centered on temporal and causal ordering, plot and action structure, and orientation toward human agents and their purposeful actions, among others, all of which are seen as text-internal and therefore pertaining to issues of form and content. The structuralists' project was a deductive, and ultimately a reductionist, method of identifying the features of narrative structure independent of the intentions or construal of the teller or reader of any story. Although classical narratologists are the main proponents of text-internal views, there is also a significant amount of psychological and early cognitive science work that similarly distinguishes narrative from other forms of thought organization on text-internal grounds. Thus, even Jerome Bruner (1986, p. 11), considered rightly the father of “folk psychology” and narrative reasoning, speaks about the “narrative” and the “logico-scientific” or “paradigmatic” modes as two distinct modes of cognitive functioning with their own specific operating principles and criteria of well-formedness that are manifestly text-specific. On his account people employ the paradigmatic type of reasoning when they think about scientific or logical matters, while narrative thought serves the purpose of explaining the changing directions of human action. Crucially, Bruner sees narrativity as a structural property, a cognitive invariant of sorts, that only later, in different discourse realizations gets a constructivist flavor. Early story grammars (Rumelhart, 1975; Mandler and Johnson, 1977) also attempted to isolate the unique internal structures (schemata) of narrative through an analogy with assumed internalized language rules believed to characterize the knowledge and use of language, as proposed by Chomsky's generative grammar. Thus, these story schemata are formalized as a set of generative rules that are used to understand and produce narrative as a specific text-type in opposition to other types such as description, argumentation or instruction. Schemata and story grammars are insufficient to explain narrative understanding on their own, however. Despite the fact that they organize aspects of memory and guide interpretation of new narratives by supplementing missing information, a good narrative is a distinctive and coherent series of events uniquely informed by a specific point of view. Despite the irreducibility of causality as a mental process, the connectivity and configuration of a good narrative are imposed by a specific narratorial viewpoint, as I will argue below, and not a result of a given narrative schema instantiation^4^.

Finally, in this group of text-internal approaches I will classify a number of theories put forward by philosophers and literary critics that have become known as poststructuralist. As an approach to the reading of fictional and other texts, deconstruction, which is another name for the poststructuralist theories I have in mind, has been the dominant paradigm for a period from the 1960's to the 1990's. Derrida's *differance* is understood as a process of dissemination of meaning wherein all communicative constraints on a producer and a receiver of meaning are removed in favor of an agentless and limitless web of signification, which works against any specific authorial intention and any given interpretation. The main thrust of the poststructuralist approaches is thus a search for the latent contradictions in texts that the participants in a communicative exchange are themselves believed to be blind to, because any intention at communicating meaning is judged to be subsumed by the discourse-driven disembodied signifying process itself. One of the reasons for this ultimately flawed understanding of language is the fact that it deliberately ignores the significant factor of meaning being born in the interaction of the meaning constitutive practices of human agents.

## Narrative as communication: text-external approaches

The set of approaches which reject the self-sufficiency of the text itself and look for describing meaning as the product of the reader's reception outnumber by far the text-internal approaches. The main dividing line concerning issues of reception has to do with the distinction between more theoretical phenomenological models of idealized, hypothetical, or universal authors/readers, and more psychologically grounded ones who have sought to explicate in a more empirically sound way some of the responses of real readers to literary texts.

A communicative understanding of literature provides the starting point for many of the text-external approaches to meaning construal in narratives. Narrative need not be always verbally instantiated but it needs to be somehow externally presented to be communicated and understood, as in a silent film, or a dance, or a mime performance. Verbal communication has been looked at in terms of the communicative intention of a speaker and the subsequent interpretation of that intention, but also in terms of existing conventions (normativity) and context. Unless some form of explicit verification of the original communicative intention is made, what gets transmitted in an act of verbal communication is a series of cues that get reconstructed by a listener. Any communicative exchange is then just an attempt at meaning making which may or may not be successful. Earlier models of communication in language relied heavily on the six elements involved in any verbal communication, proposed by Jacobson (1960), and their corresponding linguistic functions. The elements and their respective functions are: the addresser (“expressive function”); the addressee (“conative function”); the context (“referential function”); the code (“metalinguistic function”); the channel (“phatic function”); and the message (“poetic function”). Jacobson believed that all these functions are involved in every act of verbal communication but only one was dominant in any particular verbal exchange. Somewhat self-evidently, the poetic function was seen as specific to forms of verbal art, particularly poetry. What is important to note even in this early model is the realization that the message alone does not and cannot supply all the meaning of the exchange. A speech act is a process where much of what gets communicated derives from an interaction between a speaker, and a listener, but also and importantly with the help of context, code and intention. In literary theoretical approaches the shift toward understanding narrative as a form of communication has led to an increased preoccupation with understanding the reception process itself (albeit in a non-empirical way) and to a move beyond the formalism of early narratological models. In more linguistic approaches it has become evident in the increased interest in the pragmatics, rather than the semantics, of texts.

## Pragmatics, speech-act theory and relevance theory

Pragmatics, despite its close connection with linguistics, was originally developed by philosophers, such as Austin (1962) and Searle (1969), a fact that explains its preoccupation with what is taken to be the real acts and dynamic contexts of language exchanges between people. Pragmatics studies the uses of language in human communication, which have variously been termed “parole” (Saussure, 1974), “performance” (Chomsky, 1965) or aspects of “language behavior” (Lyons, 1977), and have been excluded from strict grammatical descriptions. The assumption in philosophical pragmatics is that in using language we perform various actions or speech acts, which go beyond the merely verbal exchange of words. Such an understanding of a whole narrative as a speech act is a clear precursor to more sociological views of narrative and related notions such as Labov's (2003) influential notion of “tellability” or “reportability” of a story—the reason for telling a story to somebody. The most important aspect of linguistic pragmatics for our purposes here is to recognize its open acknowledgement of some degree of cooperation and reciprocity in language understanding: meaning and understanding are always correlative. On the face of it this view appears consistent with the one being developed below of narrative understanding as participatory sense-making. The key difference is how the concept of cooperation and participation is understood: as a passive way of unpacking an intention, in the former case, or as an emergent interaction, in the latter.

One important contribution of pragmatics to narrative understanding is Grice's (1975) notion of “conversational implicature” and the related “cooperative principle,” which is nothing more than a normative assumption of cooperation between language producers and receivers in any act of verbal communication, including narrative understanding. Language is rarely able to convey meaning explicitly, so through words and sentences people say things that prompt others to make inferences and understand the implied meanings. According to Grice, four maxims, of *quantity* (is the information sufficient), *quality*, (is it true), *relation* (is it relevant), and *manner* (is it orderly), underlie the cooperative principle and give rise to different non-explicit meanings (implicatures). Thus, the successful recovery of an implicature by a recipient depends on recognition of the communicative intention of the sender. When a maxim is broken or “flouted,” this is in turn understood by the recipient to be deliberate and therefore interpreted as such. An early attempt, among others, to situate a narrative understanding within a Gricean framework is Pratt (1977), where both naturally occurring narratives and fictional narratives are seen as consistent with the maxims of quantity, relation and manner. What is specific to fictional narrative, however, is its lack of “truthfulness,” its inherent, because intended yet non-deceptive, “untruth.” This means that in telling a fictional story its author deliberately flouts the maxim of quality (its truthfulness) and thereby marks the text as a distinct form of communication. What is problematic in this description is the failure to acknowledge the relative unimportance of the reader's recognition or interpretation of this assumed illocutionary act of pretense. Does truthfulness matter for the reader's interpretation? Does the fact that fiction is in some sense not real detract from its communicative purpose or intent? Does it therefore evoke or necessitate some additional way of understanding, such as pretense or “make-believe?” This has been the position of some philosophers in the analytic tradition such as Currie (1995) and Walton (1990). In other work, Adams defines fiction as an act by an author of transferring origin to another speaker that he creates (Adams, 1985, p. 10). It is my view that emphasizing truthfulness at the expense of relevance is precisely one of the reasons why a communicative understanding of fictional narratives runs into difficulties. The lack of truth in fictional narratives is not a real problem if the principle of relevance is given the priority it deserves, a view given an extensive treatment in Walsh (2007)^5^. In other words, for narrative understanding it matters very little if the story relates real facts, but it matters a lot how it is told and how we make sense of that telling.

If the four maxims, proposed by Grice, are examined in detail it is clear that the notion of relevance is of great importance to all of them. The flouting of the maxims produces implicatures precisely because some utterances appear to be irrelevant in a given context. Some linguists have therefore argued that the *maxim of relation* (be relevant) overrides Grice's other maxims. Sperber and Wilson's (1995) *relevance theory* replaces Grice's cooperative principle with the *principle of relevance*^6^. The degree of relevance of a communicated sentence or text is dependent on two factors: context and processing effort. The optimally relevant interpretation, as defined by Sperber and Wilson, will be the least costly one in terms of processing effort and the most extensive one in the range of its cognitive and contextual effects (Sperber and Wilson, 1995, p. 125). Relevance theory rightly claims to be able to account more satisfactorily for a wider range of communication than much other modern pragmatics does. The reason for this is that it offers a psychologically valid account of the mechanisms involved in language understanding. What is psychologically realistic in this account is the acceptance that the two critical notions for relevance, context and processing effort, are psychologically motivated notions: they reflect each participant's individual and subjective assumptions about the world and the given context, not some objective, represented and pre-given versions of it. Relevance theory also emphasizes the importance of motivation, of identifying the communicator's intention, for meaning construal. At the same time, a fundamental problem for relevance theory with respect to narrative understanding is again the absence of consideration of the relational nature of that process, or, in other words, of omitting the interactional aspect of it. In assuming a single, optimally relevant and complete interpretation for all readers and all readings, relevance theory thus fails to account for the interactive, dynamic, and changeable processes of meaning construal that different readers or even the same reader engage in at different times and in different contexts^7^.

Despite the fact that pragmatic theory is useful to account for aspects of narrative understanding, along the lines described above, it has not been widely applied to narratives for that specific purpose. When it has been so applied, it has been mainly under the rubric of rhetoric. One of the best existing examples is the very influential *Rhetoric of Fiction* (Booth, 1961), where the novel, and by extension, any literary narrative, is conceived as a rhetorical act of “telling.” Booth's undeniable contribution to narrative understanding consists in elaborating on the relations existing in the narrative communicative act, and specifically the participants in it, the details of which will be discussed below. Booth's own later work (1988) develops a more interactive understanding of how readers communicate with books through his metaphor of books as friends, who can either help or harm us, thus introducing an ethical dimension to the act of communication. Other more recent attempts are the rhetorically-oriented work of Phelan (1996) and Rabinowitz (1977), both of whom also emphasize not just a communicative but an ethical dimension in the rhetorical act that is each narrative telling and reception. A step even further in literary pragmatics is understanding fictionality itself as a specific rhetorical stance, as developed by Walsh (2007). His position is that the problem of fictionality should be seen not as a problem of truthfulness, but of relevance (Walsh, 2007, p. 30) and that each narrative interpretation is ultimately a matter of how we resolve the question of relevance: why a certain text is worthy of attention, interpretation or evaluation for any given reader.

## Narrative communication: the participants

It is to some extent clear why a conversational narrative can be seen to be similar as a communicative act to other verbal exchanges like an ordinary conversation, a public speech or a letter. For that reason, in text-external approaches to narrative understanding it has been assumed that the standard for all narratives is a naturally occurring conversational narrative. Yet, it is also clear that the communicative context of a fictional narrative can be very different. For a start, any novel is a much more complex and deliberately crafted linguistic artifact than a story told at the dinner table. Secondly, the presumed intention of a writer is not available or knowable in the same way as that of a conversational participant. In early forms of practical literary criticism interpretation of texts was sought with the help of biographical or historical data on the author's life, an approach that was soon deemed flawed, however, and exposed by what is known as “the Intentional Fallacy” (Wimsatt and Beardsley, 1946). What followed was a development of a more sophisticated view of what represents an authorial intention in narrative, acknowledging that readers rely not on any actual or explicit statements of intention but, rather, recognize the indubitable assumption of intention contained in every text, a view that underlies, as I have suggested earlier, how we understand any human action.

The role of the agent(s) in any form of literary communication has been controversial and has not been resolved in any definitive way. The main disagreements concern the levels of communication in a narrative, of which there are thought to be two, although a hybrid third cross-category has been a main concern for all kinds of theoretical and practical approaches to narrative understanding. As Genette has put it, “a narrative of fiction is produced fictively by its narrator and actually by its real author (Genette, 1988, p. 139).” Yet, in the absence of a real person talking, there has been proposed another agent, a textually implied narrator or author, who leaves a mark of his/her presence on the text in the shape of its specific norms and choices^8^. The concept of the *implied author*, introduced by Booth (1961), can thus be seen to describe a text's assumed intention: an assumed agency necessarily employed when interpreting a text. The concept therefore is seen not as a simple prop in the reading process but an indispensable function of the interpretative process itself, an analytical position that every reader anticipates and fills. The controversy about the concept concerns whether it stands for some form of imagined, anthropomorphized entity or a textual process itself, with the majority of opinion weighing in on the position that the implied author is not a presence but a textual projection of the reader's own interpretative strategies. Finally, the intra-narrative level of a novel is the one where communication is taking place between a narrator, who tells the story and a narratee that may or may not be specifically mentioned. The main point that I would like to make here is that, no matter what we call it, the reader constructs some kind of a conversational participant in the process of reading, a mediating consciousness between herself and the reported events. That participant is, as Bortolussi and Dixon suggest, not an abstract or logical characteristic of the text, but a mental representation in the mind of each reader (Bortolussi and Dixon, 2003, p. 72). The narrator is a fictional, yet psychologically real, enunciating instance of an act of telling and telling is, on my view, a form of interaction. The model I am proposing below offers an explanation that need not take textual presence and anthropomorphic presence of a teller as mutually exclusive aspects of the reading process, but as constituents of the reader's co-construction of meaning in a text.

I adopt the narrator in a literary act of communication as the main participant interacting with a reader for a number of reasons. First, in naturally occurring conversational narratives, there is always a speaker. Second, literary narratives from the Homeric epic to the realist novel and beyond have a more or less explicit and sustained enunciating instance that manipulates what we get to know and how we get to know it. Indeed, for many theorists the presence of a narrator constitutes a defining feature of verbal narrative, much in the same way as a film is assumed to be shot through a camera held and manipulated by a real person. In natural narratives or nonfictional discourse the author of the discourse speaks in his or her own voice, while in fictional narratives what is said is attributed to the speaking “voice” of the text itself and originates with the narrator, an entity that is separate from the actual author (Bortolussi and Dixon, 2003; Mellmann, 2010). This is because both the implied author and narrator are identified in relation to individual texts, not a compiled entity based on many texts, something that makes them distinct from the real author. Similar descriptions include Abbott's (2002, p. 77) and Chatman's (1990, p. 77) “inferred author,” or Eco's (1990) “model author.” I hypothesize therefore that a narrator, assumed to have agency, intentionality and physical perspective is a participant in any narrative interaction with a reader^9^. If readers assume the existence of a conversational participant who is the agent responsible for the text, the process of literary interpretation is an intersubjective process of sense-making, and will be a reflection of each individual reader's distinct construction of that agent's stance. In some forms of fictional narrative, such as 1st person autobiographical fiction, there may be significant degrees of overlap between the historical author and the narrator, a fact which nevertheless does not detract from the importance of the distinction itself. What is being emphasized here is that, rather than being an “anthropomorphic fallacy,” as suggested by Bortolussi and Dixon (2003, p. 174) that participant is a real psychological effect of the interactive language processing, a symptom of the eminently social aspect of human interaction^10^. Recent neuroimaging studies have confirmed this human tendency by showing that silent reading of direct vs. indirect speech activates voice-selective areas in auditory cortex (Yao et al., 2011). Seeing narrators as ubiquitous in verbal narratives should not be seen as simply a linguistic convention or a mere form of linguistic construction (for this view see Dancygier, 2012) but a natural disposition of the inherent intersubjectivity of human minds.

Because it is ultimately a form of mental construction, there has been no unanimity in how various theorists have treated the concept of the narrator. It has been called a voice (Bal, 1985), a narrating agent (Rimmon-Kenan, 1983), a narrative position (Toolan, 1988), or some other form of inferential construction on the part of the reader (Fludernik, 1993). I suggest that the presence of a narrator underlies a specific functional feature of narrative that has already been mentioned, namely, that the goal of narrative is not primarily informative, but interactive. Narratives do not just recount general experience, but make it specific, thereby evaluating it (Polanyi, 1981), and showing it has a point that is worth sharing (Labov, 2003)^11^. If we accept that every text has a speaker and in understanding we interact with that speaker, the problem is resolved because the interactive process is not textually but contextually situated. A problem for establishing the narrator as the main participant in the interacting process may potentially be the fact that some narrators are seen as “unreliable,” that is as somebody whose rendering of the story the reader has reasons to suspect (Rimmon-Kenan, 1983, p. 100). From my perspective it is important to understand that the reader will employ whatever knowledge they have or may gain from the narrative in order to make sense of it, irrespective of the fact that they may suspect inconsistencies in the narrator's version of events. This is because the inconsistencies are there to be discovered, played with, and perhaps ultimately resolved (or not), all of which happens in the process of reading and sense-making.

## Enactive social cognitive science

Enactive approaches to human cognition foreground the social and intersubjective nature of human understanding. The name “enactive approach” to mind and life should be understood as initially proposed by Varela et al. (1991) and subsequently developed in Thompson (2007), Stewart et al. (2010) and Di Paolo and De Jaegher (2012). The most important suggestions of this approach for research on social cognition, where I situate narrative understanding, is the notion of *participatory sense-making* (De Jaegher and Di Paolo, 2007). This notion breaks with long standing assumptions about hidden intentions in individual minds, as well as with a dominant mentalistic view of how we understand others, such as “theory of mind” (Baron-Cohen, 1995). The notion of participatory sense-making captures the idea that social interactions are dynamic, unexpected, and to some extent unpredictable, hence emergent. As I have tried to demonstrate, understanding the cognitive processes involved in literary reception have followed closely what has been assumed to constitute social cognition (albeit related only to language processing), as for example, in the cases of linguistic pragmatics or discourse studies. Recently, there have been explicit attempts to describe the processes of literary interpretation as mind-reading, where reading and making sense of fiction is seen as a pleasure inducing exercise of our theory of mind (Zunshine, 2006). The problem with these approaches, as I see them, consists precisely in the mentalistic slant that they promote. While there is a more decisive turn toward exploring the socially situated nature of character minds in Palmer (2004), it is still the case that the social and public nature of mind is used here in an observer-like way to make sense of characters' actions and emotions and not as framing an interactive engagement with a reader. As Di paolo and De jaegher put it, mentalizing or reasoning about the supposed mental states of others is a legitimate cognitive process, but not one that is at play always or in general (Di Paolo and De Jaegher, 2012, p. 2). Moreover, the view that the “shared mind” is primary has been around for a long time, evidenced in the work of a number of thinkers from distinct traditions such as phenomenology (Merleau-Ponty, 1945), social-cultural psychology (Vygotsky, 1978), analytic philosophy (Hutto, 2004), developmental psychology (Trevarthen, 1979; Hobson, 2004), and more recently linguistics and cognitive semiotics (Zlatev, 2005; Zlatev et al., 2008). The enactive view of human cognition, also broadly comparable to what is called “intersubjectivity” by some theorists (Zlatev et al., 2008), proposes a markedly different view from the theory of mind positions about how we understand other people. It argues that it is not simply the case that human mental states are primarily private or solipsistic, and only subsequently, through inference or simulation, they get projected onto others so that we can know what they are thinking. The claim is that in some basic sense, forms of human engagement with others (beliefs, intentions, attentional states, and even emotions) are fundamentally intersubjective.

For theory of mind approaches there are two ways that these assumed intersubjective processes work: either through some form of information processing reliant on innate computational modules of “intention detection,” “shared attention mechanism,” etc. (Baron-Cohen, 1995) or through unconscious simulation of the intentions or feelings of another (Goldman, 2006). The implausibility and shortcomings of the former have been duly criticized by Gallagher (2008) in favor of “direct perception” in which the developing human subject engages without any need for complex mentalizing. With respect to the latter, it is of great value to look at Di Paolo and De Jaegher's (2012) own assessment of sub-personal neural mechanisms (such as mirror-neurons) that simulation theorists promote as the substrate underlying social cognition. Rather than seeing mirror mechanisms as causally responsible for social cognition (which is the dominant view), Di Paolo and De Jaegher very plausibly suggest that in fact it is interactive social experience that may produce the mirror functions and the imitative actions that are observed in human subjects. This distinction importantly draws attention to the fact that sub-personal neural mechanisms may be necessary but not sufficient for social understanding, thus depicting a crucial distinction between the two. The inherent plasticity and malleability of the mirror neuron system in humans is also indicative of social interactions playing at least an enabling role for the development of these mechanisms (Di Paolo and De Jaegher, 2012).

## Narrative enaction: changing the assumptions of narrative understanding

It is important to see the implications for social cognition of enactive cognitive science when put against the framework of embodied cognitive science as a whole. Much recent work in cognitive linguistics (Johnson, 1987; Lakoff and Johnson, 1999; Hampe, 2005) has assumed that meaning is grounded in sensorimotor experience, but this experience is commonly framed as unconscious cognitive processing as in Lakoff and Johnson's “cognitive unconscious”), basic motor schemas (Mandler, 2004; Hampe, 2005) or neural activations (Gallese and Lakoff, 2005). This framing deliberately blurs the distinction between conscious experience and sub-personal neural processes which may ultimately ground embodied experience but are not equivalent to it. Barsalou's (1999) work on perceptual symbol system, innovative as it was for its rejection of a separate abstract level of conceptual representation, also carries the mentalistic torch in equating concepts with modality-specific neural activations, thus bypassing the issue of conscious conceptual knowledge and, the social nature of its linguistic realization. Despite claims to the contrary, a description of language as essentially a private intramental phenomenon shared between people solely on the basis of their common embodiment, as promoted currently in nearly all research in cognitive linguistics, is the old mentalistic view but dressed differently. Linguistic knowledge can never be private, as Wittgenstein (1953) noted long time ago, and cannot be reduced to what goes on in individual minds or brains. The interactive nature of linguistic encounters is not addressed to a satisfactory level in the theory of “conceptual blending” (Fauconnier and Turner, 2002), where the dynamic aspect of meaning construal is noted, but human cognitive processes are described again as subconscious acts of “blending” together various elements (concepts, frames, whole scenarios), thus producing new and emergent linguistic meanings. Needless to say, none of these developments in the cognitive science of language attend to the intentional, relational, and participatory emergence of meaning among conscious subjects who share a language.

My situating of the study of narrative understanding within an enactive view of human cognition grows out of a deep dissatisfaction with various models of literary cognition, as discussed above, that have looked at narratives as texts to be interpreted, without broader considerations about how cognition is enacted. Hence, even though there are many books on cognition and narrative (Turner, 1996; Herman, 2002; Dancygier, 2012), my proposal here aims to create a more radical turn in the cognitive study of literature by firmly situating narrative study as a form of enactive cognition^12^. One of the main points that I am making throughout this paper is that stories are not static or inert cultural artifacts; they are expressions of intersubjective meaningful action and participatory sense-making between tellers (narrators) and readers. In other words, they are interactive processes in their own right, as opposed to formal structures (as assumed in structuralist narratology), or individualistic (monologic) processes of reader interpretation (as taken up in discourse studies or pragmatic theories of communication).

To bring the discussion back to narrative understanding, and specifically narrative understanding achieved through the medium of language, we need to address again the nature of linguistic meaning, but this time take into account the enactive view, as introduced above, and explore its implications for language. Particularly, it is important to look at how the inevitability of a co-evolving meaning change in any linguistic encounter can modify long-entrenched ideas about language and its nature. As shown above, traditional forms of linguistics adopt the same ontological assumption about meaning as traditional computational approaches to thought processes, namely that it is possible to analyze the world in terms of context-free data. In relation to language, this view is summed up in semantic descriptions of linguistic units as sets of fixed and independent elements, termed concepts or symbols. Pragmatics, as I have shown, attempts to override the inefficiencies of this description by postulating various contextually implied meanings, but still suffers from the assumption of a transfer model of communication between individual minds, and the accompanying assumptions of fixed predetermined meanings that require decoding. For that reason, in some accounts written and spoken language have been treated as two distinct modes of language behavior (Chafe, 1994), the former characterized as a formal system of symbols and rules; the latter, as the pragmatic use of these forms and rules in everyday speech.

This polarized view of essentially two kinds of language has been shown to be a misrepresentation and a simplification of how language works, termed “the written language bias in linguistics” (Linell, 2005). Similar view with respect to the language sciences and linguistics in general have been voiced before by Harris (1981, 1996), who suggested that linguists do not describe “real language” but fabricated, “mythical” forms of it that do not match the reality of language use. More recently, Linell (2009) has argued strongly that the dominant view in linguistics of language as a system of abstract symbols and rules that somehow get transmitted and decoded between individual minds in communication is insufficient to account for the dialogic nature of actual linguistic exchanges. He has proposed instead a view whereby the action-oriented aspects of language are given a priority and he has named this process “languaging,” as opposed to the original pragmatic term “language use” (Linell, 2009, p. 274). The latter, according to him, still promotes the abstract mental nature of language, which is then seen as secondarily and perhaps only peripherally being put to use in a given context. The process of “languaging,” on the other hand, highlights the active, spatially and temporarily situated, and interactive nature of how we speak to each other. It draws attention to the fact that meanings in language are made and not simply retrieved. It connects with the enactive view of human cognition in its recognition of the fundamentally social and co-authored nature of human meaning-making, and gives it a description unavailable in more traditional linguistic theories. A basic question concerns whether speech and writing are ultimately different in that the latter is assumed to be more complete, rigid and final, thereby restricting any potential interactive dynamics present in talk-in-interaction. The point I am making here is that when we read written narratives we enact them; we invest them with a speaker that we treat as a conversational participant, we become willing partakers in their worlds, but they also become part of ours. Narratives constitute both interventions in our sense-making powers as readers, and are, reciprocally, the dynamic constructs of the intervention itself. It is simply not true to say that narrative enaction happens in one direction only; from a text to a reader. Yes, we have all felt the unmistakable pull of a book or a film, when hours, even days and months, after reading a story a given character, a scene, or a moment stays with us to the extent that we cannot push it away. We have all experienced the inability to put a book down despite various urgent demands on our time. How does a story achieve this high level of communion with a reader? How is this possible and more importantly, why are these processes so specific to our individual sensibilities, if we take stories to be autonomous and self-contained worlds? I argue that they are not. When we read, we re-create a situation, a moment, an act in order to understand it. This understanding is shared, yet also personal and dependant on many factors such as gender, knowledge, verbal expertise, and experience, among others. Borrowing the words of the poet Antonio Machado, Varela described enaction as the laying down of a path in walking: “Wanderer the road is your footsteps, nothing else; you lay down a path in walking” (Varela, 1987, quoted in Thompson, 2007, p. 13). I would like to use the same metaphor to describe the process of literary reading: each one of us lays a path when we experience a meaningful encounter with a story. That path is and stays our own, although it may change on subsequent encounters with the same text. This uniquely subjective and experiential process that literary fiction engenders goes toward explaining the overwhelming multiplicity of interpretations that people come up with, and the consequent disagreements over literary meanings that have troubled the study of literature. This need not be considered the disciplinary disadvantage that it has been taken to be, as I will argue below.

The participatory sense-making, proposed by De Jaegher and Di Paolo (2007), pays attention to two factors: both individual cognition, and interaction, neither of which, on its own, is sufficient to account for the relational dynamics of social cognition. In the context of literary narratives this means that as readers we share in the narrating, moment by moment, of the unfolding events. Maintaining patterns of coordination, but also breakdowns of coordination and recovery are all part of participatory sense-making. I see literary narrative understanding as such a process of participation. Conflicts are possible and in fact often necessary when a particular prediction we make as readers turns out to be wrong. Narrative emotions such as curiosity, surprise, and suspense are indeed the result of such continual conflict between a reader's causal construal through trial and error of the unfolding narrative dynamics^13^. The main avenue for coordination between reader and teller in a narrative is thus temporal dynamics: flash-forwards and flashbacks in the sequence of events, the rapid tempo of a summary vs. the slowness of a scene, techniques like showing and telling, are all temporal displacements, epistemological consequences of the proximal or distal self-positioning of a narrator. A literary story, much more than the stories we tell daily, relies on how the telling decides on and arranges what is told, which the reader enacts in sense-making. This is rarely a linear process and one that leaves gaps, ambiguities, rival perspectives, and often unresolved open-endedness. Examining the interactive possibilities of telling, of mediacy in literary narratives, most commonly studied in terms of temporal/perspectival dynamics, and grammatically realized through the categories of *tense, aspect*, and *aktionsart*, thus provides a way to put side by side linguistic function and the sense-making processes of the reader. Textual features and aspects of narration, which can be studied systemically, can then be correlated with observed responses.

What I argue further is that the interactive potential of written narratives is not diminished by the nature of our encounter with them, i.e., as written texts. Linguistic choices do channel this encounter and guide the interactive process through various means, as suggested. But these are not grammatical choices only. When we enact a narratorial viewpoint, it is not because the narrator is a mere linguistic construction or a discourse feature that we decode, but because we experience it as a meaningful participatory act between ourselves and the teller. The main underlying assumption behind my claims is that the language of fiction does not simply reflect nor describe an objective reality for the reader to recreate but is very much an instrument in the co-creation, or to put in enactive terms, in the bringing forth, of that reality. If we accept, as I do, that narrative presupposes intentional directedness, a “grasping together,” which involves causality, as phenomenological narrative theorists like Mink (1978) or Ricoeur (1985) suggest, then we can say that the sense-making processes we engage in will result in a relational reshaping of that causally shaped grasping for each reader, a sense of change, of an alteration of experience^14^. This happens because so much of the experiential world of the story becomes the reader's own world.

## Narrative enaction and participatory sense-making

The enactive approach to social cognition has not been applied to literary reading in the form suggested here, although there exist a number of previous considerations, which despite using different terminology and with very different ends in mind, can be evaluated for the relational aspect of literary reading that they highlight. I examine some of these suggestions here and evaluate them in relation to the enactive view I propose, beginning with older theories and finishing with some recent ones that have relied on enactivism for their models. A theoretical focus on the reader is historically associated with the Constance School in Germany, where hermeneutics (in the case of Jauss, 1982) and phenomenology (in the case of Iser, 1978) were used to produce largely theoretical accounts for the processes of readers' contribution to textual meaning. Reception theory, as these models are known, produced some valuable contributions that can be seen as relational in the sense of enactive cognitive science. Participation is definitional to the notion of “textual gaps” or points of indeterminacy in any text (Iser, 1978), which the reader needs to fill. Literary texts have more gaps than other forms of communication, hence, require more active participation. For Iser literature is markedly different from other forms of language encounters because literary texts represent not the real and known world but generate fictive worlds which are completed in distinct ways by the reader (Iser, 1978, pp. 23–27). His main point is that textual structures, what he calls “textual perspectives” embedded in the text, in some sense control reader response, so that there are always certain limits imposed on reception processes. One significant problem for this theory is that no attempt was ever made by Iser to connect his view of the reception process with actual empirical work on real readers. At the same time, it is clear that for Iser textual meanings are understood as potentially “given” in the text and then jointly realized through reader's involvement.

Understanding the text as unfinished, as a potentiality, as a “virtual reality” has been a part of other treatments of literature that can be seen as a starting point for an enactive study of narrative texts. Ryan (2001) speaks of *immersion* in narrative worlds, Gerrig (1993) uses the metaphor of *transportation* to describe what takes place in the mind of the reader, and Nell (1988), of *entrancement* or *being lost in a book*. While these theories capture some of the reader's involvement, they still present a picture where the overall assessment of what happens with a reader in an encounter with a book is quite passive. In the analytic tradition Walton (1990) has proposed a representational theory of art, where books (and other art forms) are understood as props that prescribe and guide specific imaginings, similarly to the way children use toys to participate in games of pretense. I think that the notion of participation is already contained in Walton's view of texts as props. Given the inherent ecological meaning of props, an interesting question would be to explore types of text in relation to “easiness of use” of those props. In terms of comparison with the enactive view, Walton's is still a mentalistic view where imagination is understood as an intramental imaginary experience, instead of an interactive one. More recent views from the philosophy of aesthetics and cognitive science speak more openly of mental simulation as an important part of the reading process (Currie, 1995; Currie and Ravenscroft, 2002). Simulation is understood here as the automatic mental mimicry of a specific experience attributed to another (Goldman, 2006), hence as resulting from the sub-personal mirroring processes that simulation theories rest on. It was argued above that simulation theories of understanding other people have their serious problems, which an enactive view of social cognition tries to address. On that basis, applying simulation theories to understanding fictional minds is also problematic. Perhaps closest to the view I am proposing comes Ryan's (2001) discussion of “spatio-temporal immersion” in narrative and its connection to specific linguistic forms. Ryan rightly assumes that the reader's participation somehow relates to degrees of self-involvement (Ryan, 2001, p. 98) but these are not systematically correlated with specific textual features, and the possible dependencies remain unexplored. Ryan adopts an (unacknowledged) embodied and enactive view of making sense of a narrative when she speaks of the reader's “virtual body” inhabiting the narrative world, adopting certain perspectives, prospective vs. retrospective narration, the use of present tense, etc., all of which are taken to be specific narrative strategies for reader immersion (Ryan, 2001, pp. 133–134). It is relevant to point out here that postulating interaction, as in my proposal, instead of mental simulation gets rid of some of the difficulties faced by immersion/simulation theories.

In more recent work a prominent narratologist (Herman, 2008) has proposed an understanding of texts as a form of joint attentional engagement with artifacts. This proposal is enactive to the extent that it assumes some form of narrative intentionality which is realized not internally, as a hidden mental object to be communicated, but in the form of practical know-how whereby textual cues, for example deictic shifts, are seen as prompts (affordances) for construing meaning. While very much in agreement with the general enactive standpoint that Herman takes, I have two main reservations about this formulation. First, the accepted view in ecological psychology is that affordances are dispositional properties of physical objects^15^. Describing texts as providing affordances for interaction with an interpreter is therefore a form of sensorimotor enactivism (Hutto and Myin, 2013), more suited to explanations of practical knowledge, rather than social interaction. I am not sure to what an extent Herman takes texts to provide affordances metaphorically (at one point he compares textual designs with a coffee machine's built-in activity structure to make coffee (p. 256). If taken literally, the proposal raises a second objection in that affordances are understood here as inherent properties of texts which somehow tell us directly what to do with them, leaving the laborious and temporal process of sense-making unattended to. Yet, as I have argued before, textual understanding is a dynamic process unfolding in time, going through rhythms of coordination, breakdowns and recovery, which often does not end with a story's conclusion. The key to literary understanding, I argue, is a deliberate process of sense-making, reliant on conscious modification and regulation between intentional agents (real or imaginary), and hence necessitating a prolonged attention and also something akin to what Tomasello (2014) very recently described as “shared intentionality.” In other words, it is not the structure of narratives, or language, or culture *per se*, that generate intersubjective understanding, but the inherent socially recursive and “shared” mind that sets this process in action (see also Di Paolo and De Jaegher, 2012). Agency is prior to action and literary interpretation is continually created by readers not in the form of reproduced textual patterns (plot or structure), nor passive automatic dispositions and affordances, but as shared agency, as a constant attunement to the assumed agency of another.

Another recent view, proposed by Caracciolo (2012a), already moves beyond Herman's view of textual cues as affordances, and toward something closer to what I am proposing here. While elsewhere the author has maintained that in understanding fiction the reader simulates a fictional consciousness, most commonly the one(s) that the text gives direct access to Caracciolo (2013), here he sees narrative understanding as a dialog between author and reader, a form of shared experientiality. Despite relying on the notion of joint attention and Dennett's intentional stance (as does Herman), Caracciolo is taking a non-explicit step toward interaction when he claims that authors and readers experience a story in essentially similar ways (p. 198)^16^. Where he differs from my proposal is in his separation between experientiality (what he calls “the intentional level”), mainly seen as embodied, non-conceptual knowledge, constituting the common ground between agents in a narrative situation, and higher-order, narratively constituted interpretations, which he sees as essentially distinct from the former. The shared reality of a created storyworld is thus taken here to be based solely on the shared embodiment and shared cultural practices of the participants, and not as the shared intention of a participatory process of sense-making of individual agencies that I am proposing. As I argued above, joint attention is born in collaborative activity, that is, in shared intentionality, not just in sub-personal, shared embodiment.

## Narrative enaction: current empirical data and future possibilities

It is part of my proposal to emphasize that work done in the field of empirical studies of literature bears directly on the enactive view, as developed here. In this section I discuss the empirical possibilities of that approach, both with respect to current findings and future research. The empirical study of literature, the examination of real, as opposed to hypothetical acts of reading, is where a lot of what has been discussed above can demonstrate its validity and validation. As an experimental activity the empirical study of literature is reliant on the methods and assumptions used in psychology and discourse studies. Historically, it has been a willfully neglected field, especially given the large theoretical body of work dealing with literary meaning, as shown in the previous discussion. It is of great interest to my current proposal that some form of participatory understanding of the processes of literary reception can be found precisely among practitioners of the empirical study of literature (Bortolussi and Dixon, 2003; Miall, 2006). Bortolussi and Dixon propose an approach that they term “psychonarratology,” where textual features are examined in close correlation to reader interpretive constructions in the context of a specific reading (Bortolussi and Dixon, 2003). Miall and Kuiken (1994) and Miall (2006) investigate how specific features of the language of texts (imagery, alliteration, meter, syntactic inversion, etc.) influence meaning creation by readers.

The first main issue in empirical studies is a question of research design: how best to study a given text. Discourse studies have traditionally examined questions of inference in a text: from causal connections between narrative events, to processing of anaphoric expressions, to textual cohesion, and other text properties. This type of research uses simplified short narratives, thus greatly limiting the scope and usefulness of any findings by the assumption that all texts, regardless of complexity, make the same requirements on a reader. When real texts are the subject of experimental research, there are a number of options that researchers can take. The most promising one for participatory sense making is the one where particular aspects of a literary text are manipulated, thus isolating a specific effect, and then comparing the reception of that text with the one of the original text. If we accept the hypothesis that a reader enacts a particular narratorial consciousness, there are aspects of how the narrator is presented in a text that are immediate candidates for such empirical work. For example, 1st person, 3rd person, omniscient or figural narration require examination with respect to ease of comprehension and/or aesthetic judgment (value). Another outstanding empirical question is: do readers consciously differentiate between such types of narrators, and if they do how this influences the sense-making process? Consciousness in a novel is displaced from the situation of telling in either time (reporting the past or the future), or person (type of narrator), and these displacements correlate with specific sense-making strategies. Hence, in conversational narratives story peaks happen in the present tense and the use of the present in a literary narrative becomes a linguistic signal of immediacy vs. displacement (Chafe, 1994). Second, the long standing discussion in narratology between the two main narrative rendering techniques: *showing* and *telling* (Genette, 1980) needs to be evaluated for the same effects. Manipulation of texts with these types of specific features will provide ways to understand how the positioning of the narrator (proximal, in showing; or distal, in telling) to the narrated events affects sense-making. Again, I emphasize the point that in narrative grammatical features, like tenses, are not just forms that correspond to divisions into past, present and future, but also signals to control how some information is to be enacted. Narrating from a particular spatio/temporal or personal/vicarious viewpoint creates for the reader an experiential stance for participation in the storyworld. Third, the main narrative situations pertaining to any narrative sense-making consist of the narratological categories of person (does the narrator belong or not to the narrative world); distance (does the narrator adopt a retrospective or synchronous temporal position); and perspective (does the narrator present an inside view of events and characters, or an external one, or both) (Genette, 1980; Stanzel, 1984). The variations that these combinations provide work toward establishing degrees of availability of the narrative worlds that we inhabit as readers: as a reader I cannot conceive of an imaginary world in which I am not present. But they also serve the purpose of a reader's intersubjective alignment with the narrating consciousness of the story.

Various aspects of reader involvement have made it into the experimental designs of empirical studies. For example, Bortolussi and Dixon have studied degrees of identification with a narrator that a reader undertakes as an aspect of implicitly and explicitly given knowledge about the narrator's actions. They manipulated a text excerpt, so that it became more explicit about the narrator's purpose and created two conditions with an original and analtered text. They predicted that when the reader has to work more, as in reading the original passage, there will be more identification, more opportunity to attribute their own experience to the narrator. The results confirmed that even though the explicit altered versions provided more information, the readers saw the narrator as easier to understand in the original version. Miall's (2006) approach is also strongly consistent with the proposal of participatory sense-making. By studying “literariness” or “foregrounding,” which originates with formalist views and is traditionally associated with text-specific formal qualities such as metaphor or alliteration, Miall shows it to be a manifestation of the enhanced special nature of the interaction processes between reader and text. Literary narratives have a “dehabituating” role to play in human cognition, which means they invite us to consider frames for thought and feeling that are novel or unfamiliar (Miall, 2006, p. 3), hence more demanding. Importantly for the discussion here, dehabituation is an interactive process initiated by language forms in literary reading, but experientially correlated with heightened attentional or aesthetic states in readers that can be experimentally verified. Finally, Miall's approach points to a need to engage not just in studying how readers interpret texts but in how they experience literary works, a requirement which, importantly, includes considerations of feeling. While most theoretical and empirical work on narrative engages the issue of interpretation, an important question that remains largely unaddressed is what kind of experience is brought by reading, and the answer is emotive experience. Empirical findings about self-implication during reading (Larsen and Seilman, 1989) show that readers of literary texts draw more on active personal experience. Such results may not only be a validation of the enactive view but also a way to define what is distinctive about literature as a sense-making process.

## Conclusion

The theoretical and practical study of literary narratives has produced multiple and often contradictory ways of explaining their structure, function, and meaning. Regardless of this prolonged scrutiny there is currently no consensus as to what narratives are and why people find them both engaging and uniquely suited for expressing aspects of human experience. I have argued that stories do not happen in individual minds, either those of tellers or readers, but in the dynamic interaction between them. Traditional narratology, as well as cognitivist story grammars, have relied on static abstract structures of text which are assumed to determine readers' understanding through detached mental representations of a story world. A pragmatic communicative understanding of stories, on the other hand, has assumed that both language and the verbal stories that we tell in it, are explicable through an information processing model of cognition and a transfer model of communication, both of which have proved insufficient. I have argued that stories are best understood as processes of patterned interaction, prospectively anticipated and retrospectively reflected upon in a participatory sense-making between essentially two participants: a reader and a teller. This to some extent imaginary participant is not just a linguistic effect but a manifestation of the irreducibly intersubjective nature of human minds. Literary reading is thus a shared act of participation, moment by moment, in the unfolding action; a process of leading and being led in order to enact an experience. I, as a reader, supply the memories, the imaginings, and the feelings in order to inhabit a world that until then is not my own, but becomes my own when I enact it. A meaningful encounter with a story is thus a participatory act of performance where meaning lies not in words, concepts or events but in the intersubjective spaces they create between the participants.

### Conflict of interest statement

The author declares that the research was conducted in the absence of any commercial or financial relationships that could be construed as a potential conflict of interest.

## Footnotes

^1^See Gallagher and Zahavi (2008, p. 158). As the authors explain, this kind of conscious awareness does not have to be of a very high order; very often it is just a case of a pre-reflective awareness. At other times, there may be explicit awareness of acting for a reason, as in more complex decision making processes.

^2^Merlin Donald's theory of “mimesis” as a form of representing reality that is intentionally controlled because bodily based, goes a long way toward explaining a fundamental difference in representing reality that human beings possess in distinction to other forms of life (see Donald, 2004). Others have similarly argued that humans are unique in using the body as an instrument (a tool) for achieving understanding in the public sphere of social life where we generally dwell. (see Tallis, 2003). The main argument behind both Donald's and Tallis' proposals is that by being able to see, rehearse and refine various “mimetic skills” (Donald) or the use of the visible hand (Tallis), human beings have evolved as the embodied and enactive agents that we are, living and communicating in a public, shared and visible world.

^3^See Gallagher and Zahavi (2008, p. 7).

^4^The configurational aspect of narrative, seen as not text-internal but stemming from the act of “grasping together” has been proposed by Mink (1978) and later extensively developed by Ricoeur (1985). Mink, in particular, speaks about narrative events being properly described not just as events, but as events “under a description” (Mink, 1978, p. 145). More of this will be discussed later.

^5^In much of the psychological work on discourse processing the understanding of texts is also seen as a form of communication. This work has sought to establish how the reader is able to build and maintain a mental representation of the text world and all the actions and characters that it contains (see Van Dijk and Kintsch, 1983). What is assumed in these models, however, is a unique and unambiguous message that is encoded in the text and then decoded by any competent reader in pretty much the same way. This is a very problematic assumption for reasons that will be discussed below.

^6^The definition of the principle states that “[e]very act of ostensive (i.e., mutually manifestly intentional) communication communicates the presumption of its own optimal relevance” (Sperber and Wilson, 1995, p. 158).

^7^As a general criticism of speech act theory and other pragmatic theories of interpretation it can be said that they are, in the words of Linell (2005), “monologic” approaches to language use. This means that they fully embrace the information processing model of cognition, the simple transfer model of communication, and the code model of language, proposed as far back as Jacobson (1960).

^8^For a detailed examination of the history of the concept and its critical reception see Kindt and Müller (2006).

^9^In their comments two anonymous reviewers have raised the objection that ultimately the only minded participant in an intersubjective encounter with the reader is the real author. As I will argue below, narrative enaction is likely to depend on types of narrator as well as many other linguistic factors. Whether and how readers respond to these types of narrators remain, however, largely unexplored empirical questions, although some initial results will be discussed in the section on empirical data. It is my point that the presence of a narrator unifies and shapes the reader's response in specific ways, depending on how this imaginary participant is construed. It is possible that readers will respond differently to narrators who are named or are part of the story in some explicit way (e.g., when they are homodiegetic in Genette's, 1980 typology), as opposed to 3rd person heterodiegetic ones.

^10^For a similar view on the need for the narrator see Mellmann (2010). For the opposite view see Walsh (2007). For the view that certain types of narrative with no explicit linguistic traces of a narrator, such as 3rd person narration or narration in free indirect discourse, have no speakers, see Hamburger (1973) and Banfield (1982).

^11^It is of interest to note that the concept of the narrator has been largely ignored in studies of discourse processing. In more recent cognitive narratology the issue of intention has resurfaced with the notion of “the intentional stance,” used by Herman (2008) to account not only for what he calls “an innate tendency to read for intentions” (p. 240) in narrative practice, but also to argue that it is narrative practice itself that gives rise to such human tendency to ascribe intentionality. It is proposed by Herman that the problem of whose intention is communicated in a narrative can be resolved by treating it as a “structure of know-how” in a more general process of folk-psychological reasoning, a point to which I will return below when discussing his views on how narratives mean.

^12^I acknowledge the fact that recently there have been attempts to develop models of literary narrative understanding that also use some form of enactive cognitive science to substantiate their claims, such as Herman (2008), Caracciolo (2012a,b, 2013). The specifics of how these valuable hypotheses are situated in relation to the one proposed here will be taken up in the next section.

^13^See Sternberg's (1978) account of narrative dynamics, based specifically on these three narrative emotions.

^14^In relation to everyday story-telling a similar claim has been developed under the name of “the narrative practice hypothesis” (Hutto, 2007). The proposal is that folk-psychological understanding of other people occurs normally as an effect of story-telling practices, through the support of others. Reasons for acting thus become familiar to children through explanation, linking beliefs, desires and outcomes in social scenarios. The problem with this is that beliefs and attitudes are, more commonly, aspects of the way agents reflect, *post* hoc, on their own or others' activity. While these are verifiable in everyday contexts, explanation becomes problematic in the context of fiction.

^15^The Gibsonian sense of *affordances* (Gibson, 1979) describes an organism's perception/action in terms of the opportunities arising from its interaction with an environment. *A*ffordances are bundles not of qualitative data, but of immediately given motor information which facilitates perception and practical action (p. 134).

^16^In another paper (Caracciolo, 2012b), the author also suggests looking at narrative interpretation as a “joint process of sense-making.”
